# In Silico Modelling of the AQP0 T138R Mutation and its’ links to Potential Mechanisms of Cataractogenesis

**DOI:** 10.21203/rs.3.rs-7408454/v1

**Published:** 2025-08-29

**Authors:** Yuliia Nikolaieva, Laurel Chaproniere

**Affiliations:** Nottingham Trent University; Nottingham Trent University

**Keywords:** Aquaporin 0, T138R mutation, congenital cataracts, membrane protein stability, hydrophobicity, hydrogen bonding

## Abstract

The T138R mutation in Aquaporin 0 (AQP0), a key membrane protein in the ocular lens, causes autosomal dominant congenital cataracts. Whilst previous studies have demonstrated that this mutation disrupts water permeability and leads to protein mislocalisation, the specific structural mechanisms underlying these functional defects remain unclear. This study employed in silico approaches to characterise how the T138R substitution affects AQP0’s molecular structure and stability. Computational analysis revealed that whilst the mutation does not significantly alter the protein’s global conformation (RMSD = 0.000 Å), it may disrupt a key network of hydrogen bonds involving Glu134, Ile135, and Pro208. Multiple sequence alignment showed these interacting residues are highly conserved across species, underscoring their structural or functional importance. Hydrophobicity analysis indicated that the substitution resulted in a strongly hydrophilic, positively charged residue (Arg, Kyte-Doolittle score: −4.5) into a predominantly hydrophobic transmembrane environment. Transmembrane insertion energetics calculations demonstrated a possible increase for membrane integration (ΔGpredapp: +1.42 to + 1.63 kcal/mol), with the Arg side chain contributing nearly twice the insertion cost of Thr (+ 0.23 vs. +0.41 kcal/mol). Protein-protein interaction modelling with Connexin 50 revealed subtle but potentially significant changes at the docking interface, including potential decreased solvent-excluded surface (−0.00019646) and increased solvent-accessible surface (+ 0.00062829) changes. Additionally, potential steric clashes between Arg138 and Met183 were identified. These findings suggested reduced compactness, possible formation of internal voids and disruption of local packing. This work provided insight into the structural changes that may underlie the functional impairments of AQP0, supporting future research into its role in cataract formation.

## Introduction

Aquaporins (AQPs) are a family of membrane proteins that facilitate the passive, selective transport of water across biological membranes. They play a critical role in cellular and tissue hydration. In humans, 13 distinct aquaporins have been identified, each with specialised function and tissue-specific expression (Yang, 2023; and Verkman, 2013)

Aquaporins have a basic structure consisting of six transmembrane helical segments and two short helical segments that surround water-filled vestibules connected by a narrow aqueous pore. Aquaporin monomers assemble as tetramers in membranes, with each monomer functioning independently (Ozu et al. 2018).

The primary function of most aquaporins is to transport water across cell membranes in response to osmotic gradients created by active solute transport. The selectivity of aquaporins for water is achieved through steric factors and electrostatic interactions in the aqueous pore (Yang 2023).

Aquaporin 0 (AQP0), also known as the major intrinsic protein (MIP) of the lens, is part of the aquaporin family. It is specifically expressed in fully differentiated lens fibre cells, where it plays an essential role in maintaining lens transparency. AQP0 is encoded by the *MIP* gene located on chromosome 12q13 (Lee et al. 1996). The protein weighs approximately 28 kDa and is made up of 263 amino acids. In the membrane, AQP0 assembles as a tetramer, with each monomer acting as an individual water channel. Each monomer contains six transmembrane domains, three extracellular loops (A, C, and E), and two intracellular loops (B and D), with both the N- and C-termini facing the cytoplasm. Two conserved NPA motifs, found in loops B and E, line the narrow pore that allows water to pass through. Although AQP0 has lower water permeability than other aquaporins, it serves a dual function: regulating water flow and acting as an adhesion molecule (Kumari et al. 2013).

Cataracts — defined as opacification of the eye’s lens — are the leading cause of blindness worldwide, affecting approximately 95 million people. While age-related cataracts are the most prevalent, congenital cataracts represent a significant cause of visual impairment in children (Liu et al. 2017). Autosomal dominant congenital cataracts have been repeatedly associated with mutations in the AQP0 gene (Bateman et al. 2000; Berry et al. 2000; Francis et al. 2000; Geyer et al. 2006), highlighting the vital function of this protein in preserving lens transparency. Twelve mutations in humans have been documented (Kumari et al., 2013; Shentu et al., 2015). A notable example is a point mutation that was discovered in a five-generation Chinese family with congenital cataracts. Sequencing of the AQP0 gene showed that cysteine had substituted arginine at codon 33. This was the first documented mutation that caused complete lens opacification in AQP0’s extracellular Loop A. It is thought that this loop helps in cell-to-cell adhesion, which is crucial for maintaining the lens’s structural integrity (Kumari et al. 2013). Understanding the mechanisms by which specific mutations, lead to cataract phenotypes is essential for uncovering the structural basis of lens opacification.

The T138R mutation in AQP0 is a missense mutation (Yu et al. 2014) that replaces threonine with arginine at position 138, located within the fourth transmembrane helix embedded in the membrane (Francis et al. 2000). This substitution alters both the size and charge of the residue: threonine is a small, polar, uncharged amino acid. Arginine is significantly larger and carries a positively charged guanidinium group at physiological pH. Introducing arginine at position 138 can disrupt local protein structure by introducing steric hindrance and electrostatic interactions that are not normally present, potentially destabilising the transmembrane domain and impairing AQP0’s normal folding or function within the membrane (Kyte and Doolittle 1982; Hristova and Wimley 2010).

Clinically, individuals carrying this mutation develop progressive, multifocal cataracts, which are characterised by pinhead-sized opacities scattered throughout the lens, except the centre of the nucleus (Francis et al. 2000). These opacities worsen with age, leading to visual impairment.

Functional assays in *Xenopus laevis* oocytes demonstrated that the T138R mutation impairs AQP0-mediated water permeability. When co-expressed with wild-type AQP0, the mutant exerts a dominant-negative effect, reducing total water transport. Furthermore, confocal microscopy confirmed that the T138R-mutant protein accumulates intracellularly rather than integrating into the membrane (Francis et al. 2000). Phosphorylation plays a critical role in the trafficking of AQP0 to the plasma membrane. Treatment with the PKC inhibitor Go6979 has been shown to block AQP0 exit from the Golgi, and a Ser235Ala mutation prevents Golgi exit in overexpression models, suggesting that phosphorylation at this site is necessary for proper membrane targeting. Regulation by subcellular relocalisation has not been observed, likely due to the unique biology of lens fibre cells, which progressively lose their organelles as they mature (Markou et al. 2022). Based on the above, it has been suggested that the T138R mutation may lead to a loss of water permeability by interfering with correct trafficking of AQP0 to the plasma membrane (Yu et al. 2014).

T138R has been shown to reduce hydrophobicity within transmembrane helix 4, while other cataract-associated AQP0 mutations; such as E134G, Y177C and R187C (Kumari et al. 2013); also occur within transmembrane helices but increase local hydrophobicity at similarly conserved sites. These opposing shifts in hydrophobicity within the membrane-embedded regions of the protein suggest that both types could lead to a shared pathogenic mechanism.

As discussed, research shows that the T138R mutation affects protein folding, weakens transmembrane stability and reduces water transport. However, it does not fully explain how the mutation impacts AQP0 structure, molecular interactions or long-term lens transparency. This study aimed to investigate how the T138R mutation may alter AQP0’s local structure and thermodynamic properties, potentially compromising its transmembrane stability and function, leading to cataractogenesis.

## Methods

### Baseline structure analysis

The predicted 3D structure of wild-type AQP0 was retrieved from the AlphaFold Protein Structure Database via UniProt (UniProt Consortium 2023) (ID: P30301).

Wild-type and T138R mutant models of AQP0 were structurally aligned using the default MatchMaker command in UCSF ChimeraX (Goddard et al. 2018). The root-mean-square deviation (RMSD) between the aligned structures was automatically calculated.

### Residue Conservation Analysis via Multiple Sequence Alignment

Multiple sequence alignment (MSA) of AQP0 protein sequences was performed using the UniProt alignment tool (UniProt Consortium 2023) to assess the conservation of important residues across species. AQP0 sequences from *Homo sapiens*, *Rattus norvegicus*, *Bos taurus*, *Cavia porcellus*, *Ovis aries*, *Lithobates pipiens*, *Gallus gallus*, *Mus musculus* and *Canis lupus familiaris* were retrieved in FASTA format and aligned using default UniProt parameters.

### Local structural effects of the mutation

Hydrogen bonding patterns in the wild-type and T138R AQP0 models were analysed in UCSF Chimera 1.9 (Pettersen et al. 2004). Interactions involving Thr138 and Arg138 were examined to identify residue-specific hydrogen bond changes resulting from the mutation.

The hydrophobicity of residue 138 was assessed using ChimeraX (Goddard et al. 2018). The built-in Kyte–Doolittle scale (Kyte and Doolittle 1982) was applied to the AQP0 structure to visualise residue polarity within the transmembrane domain. Residue 138 was analysed in both the wild-type (threonine) and mutant (arginine) forms.

### Possible membrane insertion energetic

Transmembrane insertion energetics were evaluated using the ΔG predictor tool (http://dgpred.cbr.su.se) (Hessa et al. 2007). A 21-residue segment of AQP0’s transmembrane helix 4, including residue 138, was submitted in FASTA format. Separate sequences were entered for the wild-type and mutant variants.

The ΔG predictor tool (Hessa et al. 2007) was further used to generate residue-wise energetic contributions, including side-chain insertion changes and hydrophobic moment values. These were used to compare the thermodynamic parameters associated with both variants at position 138.

### Docking analysis

The STRING (Szklarczyk et al. 2023) database was used to identify interaction partners for AQP0 (MIP) and Connexin 50 (GJA8) was selected for docking analysis.

The wild-type and T138R mutant AQP0 models were manually docked with Connexin 50 using UCSF Chimera 1.9 (Pettersen et al. 2004). Initial positioning was guided by Coulombic surface colouring to identify regions with compatible electrostatic properties. Structural alignment was further informed by the predicted membrane topology and relative orientation of transmembrane domains, ensuring that docking occurred between surface-accessible regions consistent with a plausible interaction interface. The complexes were manually refined to optimise shape complementarity and minimise steric clashes. Final models were evaluated using contact point and buried surface area analysis to assess interaction differences between the wild-type and mutant forms.

### Electrostatic comparison

Electrostatic properties of the wild-type and T138R-mutant AQP0 models were assessed using the Coulombic surface colouring tool in ChimeraX (Goddard et al. 2018). Electrostatic surface representation was generated using the built-in colouring feature based on partial charges and van der Waals radii. Surface potential was visualised across the entire protein in both the wild-type and T138R mutant models.

## Results

### Hydrogen Bonding Analysis

The closest hydrogen bond with THR138 was found to be GLU134 (2.98 Å, where 1 Å = 10^−10^ metres), followed by ILE135 (3.02 Å). The furthest interaction is with PRO208, measuring 3.40 Å. The listed interactions include both side-chain hydrogen bonds and a side-chain to backbone bond. The interaction with GLU134 is formed between the hydroxyl group of Thr138 (OG1) and the oxygen atom of GLU134 (O), while the bond with PRO208 involves the hydroxyl group of Thr138 (OG1) and the carbonyl oxygen atom of PRO208 (carbonyl O) as shown in Table 1.

### Residue Conservation Analysis via Multiple Sequence Alignment

It was shown that Glu134, Thr138, and Pro208 are fully conserved with no substitutions observed in the analysed sequences. Ile135 is conserved in all species except *Gallus gallus* (chicken), where it is replaced by leucine as seen in Table 2.

### Hydrophobicity Analysis

The Kyte–Doolittle hydropathy index for the wild-type threonine at position 138 was − 0.7, indicating a mildly hydrophilic and neutral side chain (Kyte and Doolittle 1982), as shown in Table 3. In comparison, the arginine residue introduced by the T138R mutation had a much lower score of − 4.5, which reflected strong hydrophilicity and a positive charge. This change suggested that the mutation introduced a highly polar residue into the membrane environment. In ChimeraX, this difference was visualised as a shift in colour from white in the wild-type to blue in the mutant, corresponding to an increase in side-chain polarity (Goddard et al. 2018).

### Comparison of ΔG_app_ contributions

The T138R mutation results in a higher predicted apparent free energy of insertion (ΔGapp), increasing from + 1.418 kcal/mol in the wild-type to + 1.634 kcal/mol in the mutant as presented in Table 4. This indicated that the mutant helix is less thermodynamically favourable for membrane insertion compared to the wild-type.

The final predicted apparent free energy of membrane insertion(ΔGpredapp) rises from + 1.42 to + 1.63 kcal/mol, reflecting an overall reduction in membrane compatibility, as described in Table 5 The hydrophobic moment (ΔGhyd.mom.) shows a small increase from + 0.59 to + 0.63, indicating a shift toward increased polarity across the helix. Notably, the side-chain contribution at position 138 nearly doubles, from + 0.23 kcal/mol (Thr) in the wild-type to + 0.41 kcal/mol (Arg) in the mutant. This increase reflects the higher energetic cost of inserting a positively charged, hydrophilic side chain into the membrane. As a result, the total side-chain contribution (∑ΔGaa(i)app) also increases from + 1.14 to + 1.33 kcal/mol.

### Docking analysis

Contact point analysis between wild-type and T138R mutant AQP0 following docking with GJA8. While contact points remained identical (1615) between variants, the mutant exhibited a slight increase in solvent-accessible surface area (+ 0.00062829) and decreased solvent-excluded surface (−0.00019646), as seen in Table 6.

### Electrostatic Comparison

Electrostatic surface analysis revealed no detectable difference between the wild-type (T138) and mutant (R138) AQP0 models. Coulombic surface colouring methods in ChimeraX indicated that residue 138 remained neutral in both models, as illustrated in Table 7. Additionally, no changes were observed in the electrostatic potential of neighbouring residues (Leu137 and Leu139).

## Discussion

Aquaporin 0 (AQP0) is a water channel protein in the eye lens that helps maintain transparency and structure. The T138R mutation has been linked to cataracts, but its structural effects are not well understood. This study used computational tools to investigate whether the mutation disrupts AQP0’s shape, hydrogen bonding, membrane insertion or interactions with other proteins.

### Structure

Structural analysis of the wild-type and T138R mutant AQP0 models using ChimeraX’s (Goddard et al. 2018) MatchMaker tool showed no detectable conformational differences between the two structures. The root-mean-square deviation (RMSD) between 263 pruned atom pairs was calculated to be 0.000 Å, indicating complete structural overlap under the parameters tested. The alignment was performed using the Needleman-Wunsch algorithm with the BLOSUM-62 similarity matrix and secondary structure weighting (SS fraction: 0.3), confirming that the T138R substitution does not impact the overall backbone conformation or secondary structure elements under the parameters tested. These findings suggest the mutation does not significantly disrupt the overall tertiary structure AQP0, indicating that cataractogenesis is unlikely to result from major conformational changes to its three-dimensional structure.

Even though no structural changes were seen in the model, the T138R mutation might still affect how the protein assembles with other subunits. This kind of effect wouldn’t show up in a single-chain model, but it could still influence the overall structure or function. Such mechanisms are characteristic of dominant-negative mutations, which often act through disrupting multimeric assembly without destabilising the monomer. If assembly is not affected, then the lack of structural change suggests the mutation may be well tolerated. This aligns with findings that non-loss-of-function mutations often produce only mild structural effects yet still cause disease (Gerasimavicius et al. 2022).

### Hydrogen Bonding

Aquaporin’s structural integrity and function are maintained by hydrogen-bond networks formed by highly conserved residues, which stabilise the folding and oligomerisation of the protein (Kitchen et al. 2016).

Hydrogen bond analysis of the wild-type (T138) and mutant (T138R) AQP0 models revealed that the mutation could result in the loss of three specific hydrogen bonds present in the wild-type structure (Table 1). THR138 forms stabilising interactions with ILE135 (3.016 Å), GLU134 (2.981 Å) and PRO208 (3.397 Å) via its OG1 hydroxyl group. The substitution with arginine prevents these interactions from forming and reduces the overall hydrogen bond count from 253 to 250. This local loss of hydrogen bonding may compromise the structural stability of AQP0, making the transmembrane region more prone to misfolding and potentially interfering with oligomerisation or membrane insertion.

Disruption of these internal interactions can severely compromise the native conformation and functional capacity of aquaporins. For example, recent work on human AQP1 demonstrated that mutations affecting conserved hydrogen-bonding residues led to protein misfolding and impaired tetramer assembly (Drewniak et al. 2024). Similarly, in AQP2, the D150E disease-associated mutation disrupts a critical hydrogen bond between an internal loop and the C-terminal tail, triggering misfolding and endoplasmic reticulum (ER) retention (Frick et al. 2014). In AQP0, the Glu134Gly mutation disrupts a conserved hydrogen bond that aligns key backbone carbonyls in the channel, distorting the water pathway and reducing conductance (Harries et al. 2004). These findings show a general principle across the orthodox aquaporin subtype (Ishibashi et al. 2014): local side-chain hydrogen bonds are essential for proper protein folding, membrane integration and trafficking.

The T138R mutation introduces a positively charged arginine residue near Glu134, which may disrupt its proper orientation (Kozono et al. 2002). Consistent with this, the structural model of this study suggests that a stabilising hydrogen bond between Thr138 and Glu134 is lost in the mutant, supporting the idea that Arg138 could disrupt local architecture and contribute to destabilisation. Certain studies also identified Thr138 as a conserved and structurally significant residue (Harries et al. 2004). Their crystallographic model of bovine AQP0 demonstrates that Thr138 lies close to Glu134 in a region vital for orienting the pore-lining carbonyls. Substitution of Thr138 with arginine eliminates this local hydrogen bond network and introduces steric interference in a tightly packed membrane domain. It’s been proposed that this disruption distorts the arrangement of the carbonyls responsible for water coordination, thereby impairing channel function (Harries et al. 2004) and potentially contributing to cataractogenesis. The possible absence of this key hydrogen bond in the T138R mutant, as demonstrated in the present analysis, likely contributes to local structural destabilisation.

These results highlight the structural importance of Thr138-mediated hydrogen bonds in AQP0. The T138R mutation may disrupt this conserved network, supporting a mechanism of local destabilisation consistent with impaired folding and function.

### Multiple sequence comparison

Multiple sequence alignment of AQP0 orthologues revealed that Glu 134, Ile135, Thr 138 and Pro208 are highly conserved across species tested (Table 2). Notably, Pro208, Glu 134 and Thr 138 are conserved in all sequences analysed, suggesting an essential structural role, potentially in maintaining the precise geometry of the transmembrane domain near the water-conducting pore. Ile135 is also conserved in all species except for *Gallus gallus* (chicken), where it is replaced with leucine. Leucine is a substitution that retains hydrophobic character but may slightly alter side-chain geometry. The high degree of conservation of these residues highlights their likely functional significance in stabilising the local protein environment. As Glu134, Ile135, and Pro208 residues were identified as potential hydrogen bond partners of Thr138 in the wild-type model, their interactions appear structurally significant. The loss of these bonds is likely to compromise the structural stability of AQP0, potentially affecting proper protein folding, membrane integration, and trafficking.

Proline is known for disrupting the regular shape of alpha-helices because of its unique structure. It lacks the hydrogen atom needed to form normal backbone hydrogen bonds and also blocks nearby residues from forming their own bonds. As a result, when proline appears in the middle of an alpha-helix, it often breaks at least one hydrogen bond and creates a sharp bend or “kink” in the helix. Proline-induced kinks are rare, therefore their conservation in these contexts suggests that they play a crucial structural or functional role and may be essential (von Heijne 1991). In the wild-type AQP0 structure, Thr138 forms a hydrogen bond with the carbonyl oxygen of Pro208 (Table 1). This is an uncommon interaction that likely plays a specific structural role. Disruption of this bond in the mutant (T138R) version may destabilise the native conformation of AQP0, impairing its proper folding and potentially affecting oligomerisation or membrane integration. Such a structural change could contribute to the pathogenesis of cataracts.

### Hydrophobicity

**Figure 1:**
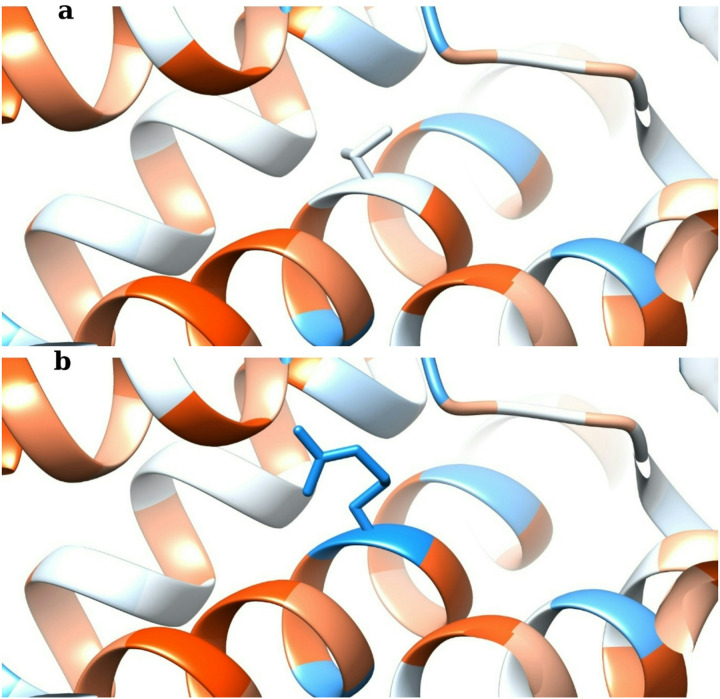
Hydrophobicity comparison of AQP0. **a** Wild-type AQP0 before mutation, showing predominantly white regions indicating neutral or moderately hydrophilic areas. **b** AQP0 T138R mutant after mutation, showing increased blue regions indicating higher hydrophilicity around the mutation site.

Hydrophobicity analysis based on the Kyte–Doolittle scale (Kyte and Doolittle 1982) showed that the wild-type residue (T138) has a hydropathy index of − 0.7, indicating a mildly hydrophilic character. In contrast, the mutant (T138R) has a significantly lower score of − 4.5, consistent with strong hydrophilicity (Table 3). The results table is based on the visual hydropathy model generated in ChimeraX (Goddard et al. 2018), in which threonine appeared white and arginine appeared blue, reflecting the increased polarity of the mutant residue (see Fig. 1). Given that transmembrane domains are predominantly composed of hydrophobic residues to enable stable membrane insertion (Stone and Deber 2017).

Arginine can be accommodated within membranes through hydration of its charged side chain and interaction with nearby lipid headgroups. This process causes local bilayer deformation and increases the energetic cost of insertion (Li et al. 2010; Hristova and Wimley 2011). In the context of AQP0 precise transmembrane packing is essential. Disruption may still impair proper membrane integration or folding, contributing to the pathogenic effects of the T138R mutation.

### Comparison of ΔG_app_ contributions

To further investigate how the change in hydrophobicity could potentially affect the protein, in silico prediction of transmembrane insertion energetics was performed using the ΔG predictor developed by Hessa et al. (2007) (Hessa et al. 2007). The apparent free energy of membrane insertion (ΔGapp), reflects the thermodynamic favorability of a protein segment, typically a transmembrane helix, integrating into the lipid bilayer, with more negative values indicating more efficient insertion. The segment analysed is a modelled 21-residue peptide centred on Thr138 of AQP0. The wild-type sequence containing threonine at this position showed a ΔGapp of 1.418 kcal/mol, while the T138R mutant showed a higher value of 1.634 kcal/mol (Table 4). ΔGapp quantifies the thermodynamic favourability of membrane integration via the Sec61 translocon, with more positive values indicating less favourable insertion (Hessa et al. 2007). Therefore, the increase in predicted insertion energy suggests that the T138R substitution may reduce the thermodynamic favourability of membrane integration for this helix.

The introduction of a charged, hydrophilic residue into an otherwise non-polar transmembrane environment could destabilise local structure, thereby reducing the efficiency of translocon-mediated membrane insertion. This is consistent with findings that charged residues within transmembrane segments can disrupt proper membrane integration and lead to retention or degradation within the endoplasmic reticulum (Harley and Tipper 1996). These findings imply that the T138R mutation may impair proper membrane embedding of AQP0, potentially contributing to misfolding or ER retention.

To gain further insight into the source of this change, ΔGapp contributions were compared between the wild-type (T138) and mutant (T138R) sequences (Table 7). The total predicted free energy of membrane insertion (ΔG_predapp)_ increased from + 1.42 kcal/mol in the wild-type to + 1.63 kcal/mol in the mutant. The side-chain contribution at position 138 increased from + 0.23 kcal/mol for threonine to + 0.41 kcal/mol for arginine, indicating that the mutation could disrupt local energetic stability. Additionally, the hydrophobic moment (ΔG_hyd.mom.)_ rose from + 0.59 to + 0.63, suggesting the helix may now be slightly less hydrophobic and more polar, which could interfere with its proper alignment within the plasma membrane or impacting its interaction with water as discussed above.

The total side-chain contribution (Σ ΔG_aa(i)app_) also increased, from + 1.14 to + 1.33 kcal/mol in the mutant. Transmembrane helices must be highly hydrophobic because the energetic cost of inserting the helical backbone into the membrane is only balanced by favourable interactions from hydrophobic side chains (Cymer et al 2015). In addition, minimally hydrophobic helices are more likely to adopt partially inserted, metastable states, which may predispose them to degradation if they fail to remain fully embedded in the bilayer (Lu et al., 2018).

The increase in hydrophobic moment in the T138R mutant could cause reduced hydrophobicity, suggesting poorer side-chain compatibility and greater energetic cost during insertion. These potential changes align with consistent established models of membrane insertion, which show that even modest increases in ΔGapp (particularly those resulting from the introduction of charged residues like arginine) can significantly reduce the efficiency of translocon-mediated integration and destabilise transmembrane helices (Cymer et al. 2015).

Overall, the docking findings suggest that although the T138R mutation may not overtly disrupt the global interaction surface with Connexin 50, it still could cause subtle structural changes. Such alterations may compromise AQP0’s membrane integration and structural stability within the lens.

### Docking

Based on the STRING protein–protein interaction network, GJA8 (Connexin 50) was identified as a high-confidence interaction partner of MIP (AQP0) and is suggested to be co-expressed with it in lens fiber cells (Gu et al. 2019). As such, Connexin 50 was selected for docking analysis to evaluate the potential structural impact of the T138R mutation in AQP0 on this interaction. Loops A and C were selected as primary docking interfaces on AQP 0 due to their established role in adhesive interactions (Kumari et al. 2019).

Docking simulations showed the number of contact points between AQP0 and Connexin 50 remained unchanged at 1615 for both the wild-type and T138R-mutated complexes, suggesting that the overall interaction surface should be retained. While docking does not precisely capture the physiological depth or alignment of membrane protein interfaces, the unchanged contact count supports the interpretation that the mutation does not significantly alter the gross interaction interface between the two proteins.

Analysis of surface properties proposed subtle changes (Table 6). The solvent-accessible surface area (SAS) became less negative in the mutant (increasing by 0.00062829), indicating potential increased exposure to solvent, consistent with the larger arginine side chain. This trend is consistent with previous studies showing that substituting bulky or polar residues into buried regions may destabilise the local structure by creating an environment incompatible with the residue’s physicochemical properties, potentially altering solvent accessibility through local unfolding or rearrangement (Dehouck et al. 2006). Simultaneously, the solvent-excluded surface (SES) became more negative (decreasing by 0.00019636), and the accompanying loss of hydrogen bonds suggests a possible local reduction in packing density or the formation of loosely packed regions within the protein structure. As illustrated by Daberdaku and Ferrari (2016) this combination of decreased SES and increased SAS suggests an expansion of the probe-accessible region between the two surfaces. This increased probe sphere accessibility could be an indicator of a more porous protein structure where solvent molecules can potentially interact with previously inaccessible regions. Such structural changes could lead to the formation of internal voids or cavities within the T138R mutant.

A similar relationship between internal cavities and reduced structural stability was observed by Eriksson et al. (1992), who showed that cavity-creating mutations result in persistent voids and destabilisation due to the loss of van der Waals interactions. This suggests the mutant protein is less compact, with larger surface irregularities and possibly internal voids. Similar structural consequences have been observed in classic cavity-creating mutations, where internal packing disruptions led to measurable destabilization (Eriksson et al. 1992). Such features may weaken intramolecular interactions and contribute to local destabilisation. This interpretation is further supported by the possible loss of stabilising hydrogen bonds in the T138R variant, reinforcing the hypothesis that this mutation could disrupt internal architecture in a manner consistent with functional impairment.

**Figure 2:**
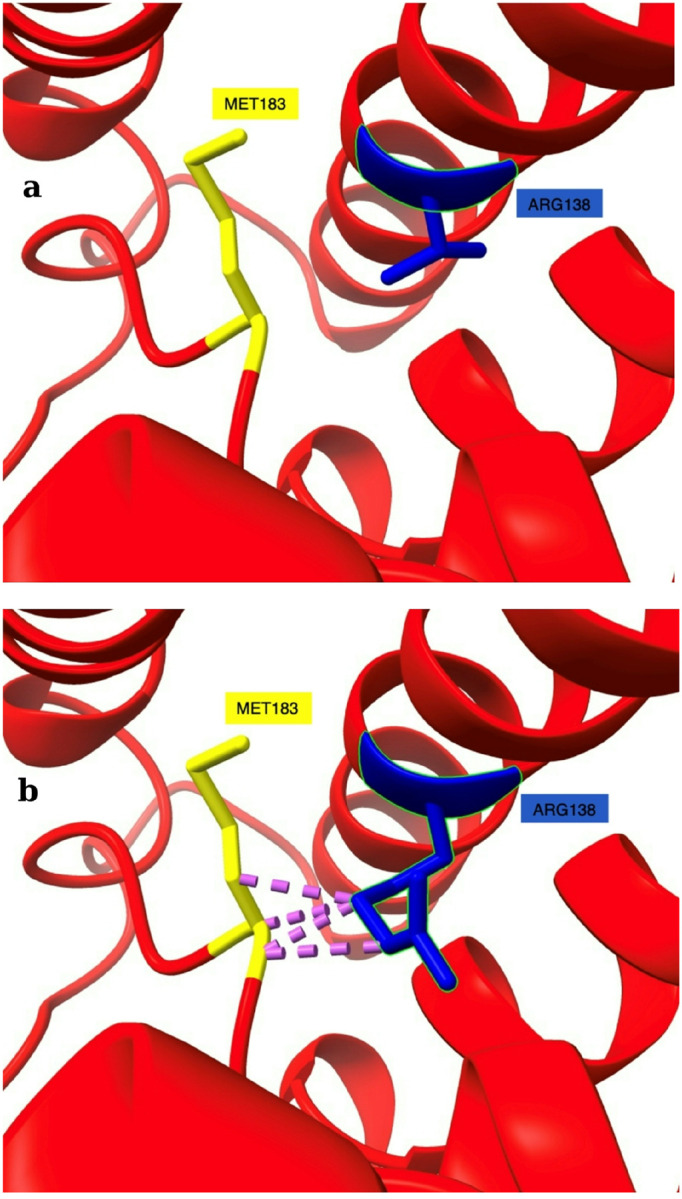
Structural and interaction analysis of wild-type and T138R mutant AQP0 in complex with GJA8. **a**Wild-type AQP0 showing normal interaction with GJA8. **b** Close-up view of the T138R mutant, where ARG138 (blue) clashes with MET183 (yellow), with van der Waals overlaps (pink) at distances of 2.204 Å and 2.778 Å. These steric clashes may disrupt proper protein–protein interaction

**Figure 3:**
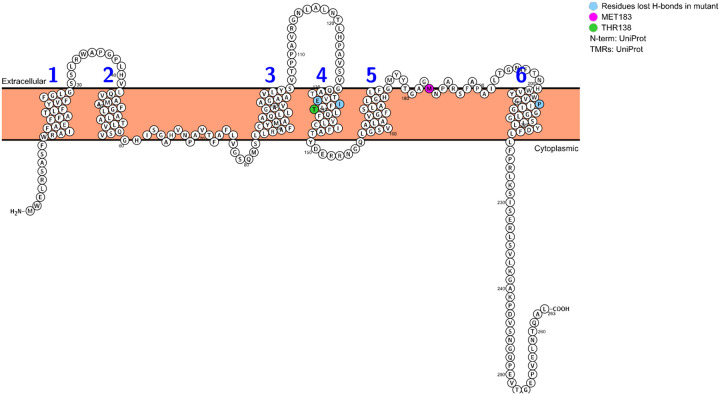
**Transmembrane topology of AQP0** visualised using Protter, showing key residues and orientation The diagram displays the transmembrane arrangement of AQP0, with light blue markers indicating residues predicted to lose hydrogen bonds in the T138R mutant. The mutation site Thr138 (green) and nearby residue MET183 (pink) are highlighted to show potential steric clash. N-terminal and C-terminal (COOH) ends are labelled, with residue numbers indicating sequence position. Extracellular and cytoplasmic regions are marked to illustrate membrane orientation (Omasits et al. 2014)

Structural analysis of the mutant further revealed 8 steric clashes between ARG138 and MET183, with van der Waals overlaps as close as 2.204 Å (Fig. 2A). Notably, clashes were observed between A/MET 183 HA and ARG 138 CD at distances of 2.204, 2.778, and 2.782 Å. THR138 is located on the fourth transmembrane helix (TM4), embedded in the middle of plasma membrane, while Met183 resides in the membrane-embedded half-helix following the fifth transmembrane segment (TM5), positioning it adjacent to the extracellular space (see Fig. 3). In the T138R mutant, the spatially demanding, positively charged arginine side chain may interact unfavourably with the partially extracellular Met183. Together with other findings of this study, the increase in hydrophobic moment and ΔG_app_ following the T138R substitution supports the conclusion that this charged residue could introduce an energetic and structural mismatch with the plasma membrane, potentially acting as a local clash that disrupts helix-lipid interactions and impairs membrane integration (Hessa et al. 2007; Cymer et al. 2015). Given that Met183 is located near the extracellular boundary of the membrane, additional effects on local structure or solvent exposure are possible; however, these potential consequences were not assessed in this study and remain to be experimentally verified.

Although several steric clashes were detected, the shorter 2.204 Å distance likely reflects a potential significant structural issue. Clashes at 2.778 Å and 2.782 Å represent smaller van der Waals overlaps and may be energetically tolerated, as such minor clashes are commonly observed even in high-resolution structures (Word et al. 2010).

### Docking (Rotamer selection)

By default, Chimera applied a low-prevalence rotamer of arginine at position 138 (χ angles: − 68.7, − 73.6, − 68.1, 109.0; prevalence: 0.001403). This conformation was likely selected for structural visualisation due to its minimal steric clashes within the local environment. For comparison, the most statistically common rotamer (prevalence: 0.165612; χ angles: − 69.2, 179.5, − 179.0, 170.6) was manually applied and modelled as well. However, this conformation introduced multiple steric clashes with MET183 and PRO208.

Notably, MET183 clashed with both tested rotamers of Arg138, raising the possibility that the substitution introduces persistent steric interference in this region. These findings support the idea that the T138R mutation could disrupt local packing and may destabilise transmembrane helix interactions, regardless of the side-chain conformation adopted.

Although the selected rotamer has low overall prevalence in structural databases, it appears most energetically favourable in the AQP0 local environment. Its conformation is supported by the absence of significant steric clashes with surrounding residues, unlike the more common rotamer, which introduced clear structural conflicts. This highlights how functionally relevant residues in membrane proteins may adopt rare but stabilising conformations in membrane proteins.

### Electrostatic effects

The electrostatic effects of the T138R mutation were analysed using Coulombic colouring in UCSF Chimera 1.9 (Pettersen et al. 2004); however, no visible changes were observed (Table 7. Figure 1). This absence of detectable change is likely attributed to the internal positioning of the mutation within the pore. However, previous electrostatic modelling studies highlighted that surface potential maps often emphasise solvent-accessible regions, while buried or internal electrostatics may be underestimated unless specifically targeted using high-resolution calculations (Baker et al. 2001). Nevertheless, both models tested exhibited a uniformly neutral (white) external surface in the region surrounding residue 138, suggesting no significant electrostatic shifts at the mutation site (Table 7). Additionally, analysis of neighbouring residues, including Leu137 and Leu139, revealed no discernible electrostatic differences between the wild-type and mutant models. These results indicate that the substitution of neutral threonine with positively charged arginine most likely doesn’t produce electrostatic changes. Unless they were not detectable by surface-rendered methods in UCSF Chimera 1.9. As a result, the mutation is unlikely to affect how this region of the protein sits within the plasma membrane.

Nemeth-Cahalan and Hall (2000) established that AQP0 undergoes pH-sensitive gating via protonation of surface-exposed histidine residues, particularly His40. This reversible electrostatic change enables the channel to close at low pH. In contrast, the T138R mutation is located within the fourth transmembrane helix, buried in the membrane interior, and is unlikely to be associated with any pH-sensitive gating domains. Since electrostatic surface analysis showed no visible potential change near this region, the T138R mutation is unlikely to influence pH-sensitive gating, and this regulatory mechanism is unlikely to contribute to its pathogenic effect.

#### Limitations

While this study provides valuable insights into the structural and electrostatic consequences of the T138R mutation in AQP0, several limitations must be acknowledged. All analyses were conducted using in silico modelling approaches, including ChimeraX/UCSF Chimera 1.9-based Coulombic colouring and ΔGapp predictions (Hessa et al. 2007), which, while informative, do not fully capture the complexity of native membrane environments. For instance, Coulombic surface colouring provides a static and simplified electrostatic representation that may not reflect the dynamic influence of lipid bilayers or neighbouring proteins, especially in deeply embedded regions such as the transmembrane site where Arg138 resides (UCSF Chimera n.d.). Furthermore, no molecular dynamics (MD) simulations were performed to assess how the mutation might alter the conformational flexibility, hydrogen bonding network, or water permeability of AQP0 over time. Incorporating MD simulations would allow investigation of how transient structural changes contribute to the observed functional deficits.

Experimental validation is essential to confirm the physiological relevance of these computational predictions. Although previous functional assays in *Xenopus laevis* oocytes have demonstrated that the T138R mutation reduces water permeability and leads to intracellular retention of AQP0 (Francis et al. 2000), these findings do not explain the precise structural mechanisms driving this mislocalisation. Future experiments could involve trafficking assays in mammalian cell lines, introducing the T138R mutation alongside other targeted substitutions to assess whether disruption of hydrogen bonding at Thr138 or steric clashes with Met183 underlie the trafficking defect.

Additionally, thermal shift assays could help evaluate the mutation’s impact on protein stability by comparing the melting temperatures of wild-type and T138R AQP0, revealing whether the substitution affects global folding or conformational integrity (To and Torres. 2015). Proteoliposome reconstitution assays could also be employed to directly measure water permeability and membrane insertion efficiency under defined lipid compositions, offering a controlled platform to isolate the functional impact of the mutation (Steffen et al. 2022). These in vitro approaches would provide complementary evidence to support structural hypotheses derived from modelling. Moreover, microscale thermophoresis (MST), a sensitive method using minimal sample and tolerant of detergents, has been specifically employed to characterize interactions between human aquaporins and soluble partners under controlled conditions (Al-Jubair et al. 2022).

Live-cell imaging methods such as fluorescence recovery after photobleaching (FRAP) have been used to study the supramolecular assembly and mobility of aquaporins like AQP4 in real time, making this technique suitable for investigating AQP0 membrane dynamics (Rossi et al. 2011). Similarly, total internal reflection fluorescence (TIRF) microscopy enables selective visualisation of aquaporins at the cell surface and has been used to monitor AQP4 membrane diffusion and clustering behaviour, providing a basis for comparable localisation studies of AQP0 (Crane, Tajima and Verkman 2009).

## Conclusion

This research suggests potential important changes induced by the T138R mutation in AQP0, based on in silico structural characterisation. Despite no detectable global conformational changes in the backbone structure, detailed analysis indicated that the mutation may cause localised structural disruptions, particularly the loss of key hydrogen bonds with Glu134, Ile135 and Pro208. These interactions could be crucial for maintaining proper folding and oligomerisation of AQP0, and their disruption may destabilise the native conformation and impair membrane integration. Further modelling indicates that the mutation may introduce a steric clash with Met183 and increase local hydrophilicity and insertion energy, supporting the idea that Arg138 creates an energetically unfavourable environment within the membrane. Docking simulations revealed subtle but potentially meaningful changes at the AQP0–Connexin50 interface, including altered solvent exposure and reduced packing density, which may contribute to functional impairment. Although previous in vitro studies have demonstrated the functional consequences of the T138R mutation, this work provides suggestions for a potential mechanistic explanation at an atomic level, linking specific structural alterations to protein misfolding and loss of function. This work also provides insight into the structural disruptions that may contribute to functional impairments of AQP0, thereby supporting future research into its role in cataract formation.

## Figures and Tables

**Table 1:** Hydrogen bonds formed by Thr138 in wild-type AQP0 and lost in the T138R mutant (Pettersen et al. 2021)

Wild-type (T138) Interaction	Bond Type	Distance (Å)
THR138 – ILE135 (OG1–O)	Side-chain H-bond	3.02
THR138 – GLU134 (OG1–O)	Side-chain H-bond	2.98
THR138 – PRO208 (OG1–Carbonyl O)	Side-chain to backbone	3.40

**Table 2:** Conservation of key AQP0 residues involved in hydrogen bonding with Thr138 across species (Sievers et al. 2011)

Residue	Conservation Across Species	Substitution Observed
Glu134	Fully conserved	None
Ile135	Conserved in all species except *Gallus gallus*	Replaced by leucine in *Gallus gallus*
Thr138	Fully conserved	None
Pro208	Fully conserved	None

**Table 3:** Kyte–Doolittle hydropathy scores at residue 138 for wild-type and T138R mutant AQP0 (Kyte and Doolittle1982)

Variant	Residue at Position 138	Kyte-Doolittle Score	Hydropathy Interpretation	Colour in Visual Model
Wild-type (T138)	Threonine (T)	−0.7	Mildly hydrophilic (Neutral)	White
Mutant (T138R)	Arginine (R)	−4.5	Strongly hydrophilic (Charged)	Blue

**Table 4:** Peptide Sequence and Apparent Free Energy of Insertion (ΔG_app_) for Wild-type (T138) and Mutant (T138R) AQP0 (http://dgpred.cbr.su.se) (Hessa et al. 2007).

Sequence	Variant	ΔG_app_ (kcal/mol)
VEIFLTLQFVLCIFATYDER	Wild-type (T138)	+ 1.418
VEIFLTLQFVLCIFARYDER	Mutant (T138R)	+ 1.634

**Table 5:** Residue-Wise ΔG Contribution Breakdown for Wild-Type and Mutant AQP0. Presented data breaks down the contributions to this difference (http://dgpred.cbr.su.se) (Hessa et al. 2007).

Category	Wild-type (T138) (kcal/mol)	Mutant (T138R) (kcal/mol)
ΔG_predapp_ (Final)	+ 1.42	+ 1.63
ΔG_hyd.mom.(_Hydrophobic moment)	+ 0.59	+ 0.63
Side-chain contribution (position 138)	+ 0.23 (Thr)	+ 0.41 (Arg)
Σ ΔG_aa(i)app_ (Total side-chain)	+ 1.14	+ 1.33

**Table 6:** Contact point, SAS and SES values for wild-type and T138R AQP0 following docking with GJA8 (Goddard et al. 2018).

Parameter	Wild-type (T138)	Mutant (T138R)	Difference
Contact points	1615	1615	-
Solvent-Accessible Surface (SAS)	−0.00435761	−0.00372932	+ 0.00062829
Solvent-Excluded Surface (SES)	−0.00179146–0.00179146–0.00179146–0.00179146	−0.00198792	−0.00019646

**Table 7:** Electrostatic Surface Comparison of Wild-Type and T138R AQP0 Models (Goddard et al. 2018).

Analysis Method	Wild-type (T138)	Mutant (T138R)	Electrostatic Difference
Coulombic surface colouring	Neutral	Neutral	None
Neighbouring residues (Leu137&139)	Neutral	Neutral	None
